# Macrophage-Specific NF-κB Activation Dynamics Can Segregate Inflammatory Bowel Disease Patients

**DOI:** 10.3389/fimmu.2019.02168

**Published:** 2019-09-11

**Authors:** Stamatia Papoutsopoulou, Michael D. Burkitt, François Bergey, Hazel England, Rachael Hough, Lorraine Schmidt, David G. Spiller, Michael H. R. White, Pawel Paszek, Dean A. Jackson, Vitor A. P. Martins Dos Santos, Gernot Sellge, D. Mark Pritchard, Barry J. Campbell, Werner Müller, Chris S. Probert

**Affiliations:** ^1^Department of Cellular and Molecular Physiology, Institute of Translational Medicine, University of Liverpool, Liverpool, United Kingdom; ^2^Faculty of Biology, Medicine and Health, School of Biological Sciences, University of Manchester, Manchester, United Kingdom; ^3^LifeGlimmer GmbH, Berlin, Germany; ^4^Department of Agrotechnology and Food Sciences, Wageningen University, Wageningen, Netherlands; ^5^University Hospital RWTH Aachen, Aachen, Germany

**Keywords:** inflammatory bowel disease, NF-κB, macrophages, cytokines, Crohn's disease, ulcerative colitis

## Abstract

The heterogeneous nature of inflammatory bowel disease (IBD) presents challenges, particularly when choosing therapy. Activation of the NF-κB transcription factor is a highly regulated, dynamic event in IBD pathogenesis. Using a lentivirus approach, NF-κB-regulated luciferase was expressed in patient macrophages, isolated from frozen peripheral blood mononuclear cell samples. Following activation, samples could be segregated into three clusters based on the NF-κB-regulated luciferase response. The ulcerative colitis (UC) samples appeared only in the hypo-responsive Cluster 1, and in Cluster 2. Conversely, Crohn's disease (CD) patients appeared in all Clusters with their percentage being higher in the hyper-responsive Cluster 3. A positive correlation was seen between NF-κB-induced luciferase activity and the concentrations of cytokines released into medium from stimulated macrophages, but not with serum or biopsy cytokine levels. Confocal imaging of lentivirally-expressed p65 activation revealed that a higher proportion of macrophages from CD patients responded to endotoxin lipid A compared to controls. In contrast, cells from UC patients exhibited a shorter duration of NF-κB p65 subunit nuclear localization compared to healthy controls, and CD donors. Analysis of macrophage cytokine responses and patient metadata revealed a strong correlation between CD patients who smoked and hyper-activation of p65. These *in vitro* dynamic assays of NF-κB activation in blood-derived macrophages have the potential to segregate IBD patients into groups with different phenotypes and may therefore help determine response to therapy.

## Introduction

Inflammatory bowel disease (IBD) is characterized by an imbalanced immune response, leading to a pro-inflammatory phenotype with elevated tissue concentrations of various cytokines including tumor necrosis factor (TNF), interleukin-6 (IL-6), and interferon-γ (IFNγ) (1, 2). One of the key mechanisms involved in generating this inflammatory environment within the intestinal mucosa is the activation of the transcription factor nuclear factor kappa-light-chain-enhancer of activated B cells (NF-κB) (3). The NF-κB family of transcription factors contains five subunits, p65 or RelA, p50, c-Rel, p52, and RelB, that may function as homo- or hetero-dimers. These dimers are retained in an inactive state in the cytoplasm by binding to a member of the family of inhibitory κB proteins (IκBs). Upon stimulation, IκBs are degraded, and the NF-κB active dimers translocate into the nucleus where they regulate transcription. The nuclear translocation of NF-κB proteins is a highly regulated, dynamic event, characterized not only by the transport of NF-κB dimers into the nucleus following stimulation, but also by shuttling between the nucleus and cytoplasm of the cell, with context-specific oscillatory frequency; such as has been observed for transcriptionally active p65 subunit dimers (4). To date, these heterogeneous dynamics have been demonstrated using mouse (5–7) and human cell-lines (8), and primary cells obtained from transgenic mice expressing fluorescent fusion proteins (9, 10).

NF-κB activation and dysregulated cytokine production has previously been reported in various cell types in IBD patients (11, 12). Macrophages and epithelial cells isolated from inflamed intestinal biopsies showed augmented levels of NF-κB (13). Lamina propria fibroblasts have been reported to be involved in cytokine production due to highly activated p65 (14), and epithelial NF-κB signaling has been implicated in several murine models of IBD (11, 15, 16). Inevitably, the NF-κB pathway has become an attractive target for therapeutic interventions in IBD, and many of the current drugs that are used to treat IBD either directly or indirectly influence NF-κB signaling (e.g., corticosteroids, anti-TNF monoclonal antibodies, and 5-aminosalicylates). Nevertheless, a significant proportion of patients do not respond to these treatments (17–19). The reasons for treatment failure are not completely understood. In clinical practice, there is an increasing use of therapeutic drug monitoring to characterize treatment failure, but, even in carefully observed cohorts, the development of anti-drug antibodies can only explain a small proportion of secondary losses of response to therapy and does not explain primary failure to respond (20). There is, therefore, a need for more specific laboratory tests to better stratify patients for therapy prior to drug initiation.

We hypothesized that patients with different clinical phenotypes would have differences in their NF- κB activation responses. Previous studies looking at NF-κB activation in human samples have largely relied on static measurements of DNA-binding activity using electrophoretic mobility shift assays (EMSAs) (21). Whilst EMSAs clearly demonstrate NF-κB DNA binding, they do not demonstrate transcriptional efficiency, and are thus unable to illustrate the dynamics and heterogeneity of NF-κB activation. Hence, in this study, we describe a novel screening protocol based on the dynamic detection of endogenous NF-κB activation in human peripheral blood mononuclear cell-derived macrophages. We report the characteristics of this assay, including its reproducibility, and its ability to segregate individuals into different clusters. Changes in NF-κB dynamics correlated with differences in macrophage cytokine secretion response and we report how this reflects the clinical phenotype of individuals.

## Methods

### Human Study Design—Ethics Approval

We performed a study using a total of 65 healthy donors and IBD patients from the UK and Germany. In Liverpool (UK), patients attending for colonoscopy at the Royal Liverpool and Broadgreen University Hospitals NHS Trust (RLBUHT) for any clinical indication were recruited. At University RWTH Aachen (Germany), only individuals with an established diagnosis of IBD were recruited. Patient and healthy control blood samples and intestinal tissue biopsy specimens were obtained following informed consent and with study approvals from NRES Committee North West-Liverpool East (R&D 4910; REC 15/NW/0045) and the Regional Human Ethics Committee, Aachen, Germany (EK 235/13). An additional cohort of 14 patients were recruited at the RLBUHT, who donated fresh peripheral blood, from which PBMDMs were generated, for confocal imaging of NF-κB activation.

### Mouse Strains

The *Nfkb1*^−/−^ mice (22) were bred in a specific-pathogen-free environment at the University of Liverpool, and all experiments were performed in accordance with regulations of the Home Office of the United Kingdom (PPL: 70/8457).

### Lentiviral NF-κB Transcriptional Activity Vectors

NF-κB transcriptional activity was monitored using a lentiviral construct (κB-NLSluc) that expresses firefly luciferase under the control of the classical NF-κB promoter. This construct is a reporter for NF-κB (5κB-Luc) in which the 5x repeat κB consensus sequence was introduced into the luciferase vector using Pac1 and Nhe restriction enzymes (23). For confocal imaging, the human p65 sequence was amplified from p65-dsRedXp (24) and C-terminally fused with AmCyan protein using a previously described lentiviral vector (9). Lentivirus production was carried out as previously described (25).

### Human Peripheral Blood Mononuclear Cell-Derived Macrophages—Isolation, *in vitro* Differentiation and Transduction

Peripheral venous blood (10 mL) was immediately heparinized (unfractionated heparin sodium, at 5 U/mL; Wockhardt UK Ltd; Wrexham; Wales). Each sample was mixed 1:2 with sterile phosphate-buffered saline pH7.3 (PBS), layered over 20 mL Ficoll-Paque™ plus (Thermo-Fisher Scientific; Paisley, UK) and centrifuged at 400 × g for 40 min at room temperature. Peripheral blood mononuclear cells (PBMCs) were aspirated, washed with sterile PBS, resuspended in 1 mL freezing medium [88%v/v FCS (Sigma, Poole, UK) plus 12%v/v DMSO (Sigma)] and stored at −80°C. Frozen isolated PBMCs were thawed and plated (4 × 10^6^ cells/well) in 6-well plates (Nunclon Vita surface; Thermo Fisher Scientific) in 3 mL differentiation medium [RPMI-1640, 10%v/v FCS, 10 mM HEPES (Sigma), 1 mM sodium pyruvate (Thermo Fisher Scientific), 1X MEM non-essential amino acids (Thermo Fisher Scientific), 10 U/mL penicillin, 10 mg/mL streptomycin, 2 mM L-glutamine (Sigma), and 50 ng/mL human macrophage colony-stimulating factor (Peprotech; London, UK)]. On day 1, non-adherent cells were washed, and fresh differentiation medium was added. On day 4, 3 ml fresh medium was added into the cultures which were incubated for further 3 days. Following differentiation, 70–80% of the adherent macrophages (peripheral blood mononuclear cell-derived macrophages, PBMDMs) expressed characteristic macrophage cell-surface markers (CD11b^+^, CD14 low). PBMDM cultures were infected with κB-NLSluc lentivirus on day 4 after 24 h the medium was replaced with fresh medium and the cultures were incubated for further 72 h. The volume of the lentivirus used was optimized per virus batch to achieve the highest level of transduction without causing cell death.

### Murine Bone Marrow-Derived Macrophages—*in vitro* Differentiation, Transduction, and Luciferase Assay

Bone marrow-derived macrophages (BMDM) were prepared as described previously (26). Briefly, bone marrow cells were plated in 10 mL of differentiation medium [RPMI-1640 (Sigma) supplemented with 10%v/v FCS, 50 mM granulocyte-macrophage colony-stimulating factor (M-CSF; PeproTech), and 50 μM β-mercaptoethanol (Sigma) at 5 × 10^6^ cells per 90-mm bacterial petri dish (Sterilin Ltd; Newport, UK). After 4d, the adherent cells were harvested, counted and plated as 1 × 10^6^ cells per well in a 6-well plate in 0.8 mL differentiation medium containing an appropriate amount of lentivirus. After 24 h the medium was replaced with fresh medium and the cultures were incubated for further 72 h. For the luciferase assay, cells were cultured in 24-well plates (OptiPlate-24, White Opaque 24-well Microplate; PerkinElmer) in 0.2 mL medium containing 1 mM luciferin (Promega; Southampton, UK). Cells were stimulated with 10 ng/mL lipopolysaccharide (LPS) derived from *Salmonella enterica* serovar Minnesota R595 (Enzo Life Sciences; Exeter, UK) and luminescence was detected over time in a CO_2_ Lumistar Omega luminometer (BMG Labtech; Ortenberg, Germany).

### Human Peripheral Blood Mononuclear Cell-Derived Macrophages—κB-NLSluc Luciferase Assay

Frozen PBMCs isolated from peripheral venous blood were thawed and differentiated to PBMDMs, as described earlier. Transduced PBMDMs were cultured in 24-well plates (OptiPlate-24, White Opaque 24-well Microplate) in 0.4 mL medium containing 1 mM luciferin. Cells were stimulated with 200 ng/mL LPS and luminescence detected over time in a CO_2_ Lumistar Omega luminometer. Post-assay, pro-viral copies were measured by qPCR, using a Lenti-X™ Provirus Quantitation Kit (Clontech; Oxford, UK). For *in vitro* stimulation, other ligands used included human recombinant Interleukin-1β [IL-1β](PeproTech), Flagellin FliC from *Salmonella* typhimurium (NovusBio; Littleton CO, USA), muramyl-dipeptide [MDP] (InvivoGen; Toulouse, France), and LPS extracted using modified phenol/water method (27) from IBD mucosa-associated *E. coli* isolates, LF82 and LF10 (28, 29).

### Confocal Imaging of p65-AmCyan Lentivirus-Transfected Human Peripheral Blood Mononuclear-Derived Macrophages

Fresh PBMCs were obtained from whole blood taken from patients recruited in Liverpool. PBMCs (4 × 10^6^ cells) were plated in 35 mm μ-plate imaging dishes (Ibidi GmbH; Martinsried, Germany) and differentiated as described above. On day 4, macrophages were infected by addition of p65-AmCyan lentivirus into the culture medium. After 72 h incubation, media was removed, fresh medium containing supplements (but no M-CSF) added and cells were imaged 24 h later. Cells were stained for 1 h with 10 ng/mL Hoechst 33342 (Sigma), medium changed and cells then rested for 1 h prior to imaging. Cells were imaged using a Zeiss LSM880 confocal microscope system equipped with a cell incubation unit maintained at 37**°**C, in a humidified atmosphere of 5% CO_2_. p65-AmCyan nuclear fluorescence was detected (excitation λ 458 nm, emission λ 489 nm), and quantified using CellTracker software (30). Basal readings were obtained for 30 min and cells then stimulated with 200 ng/mL endotoxin Lipid A (Sigma). Cells with a high starting variance in the absence of stimulation (standard deviation >10) were excluded from analyses. All measurements performed 200 min after stimulation were also excluded. Graphs were analyzed based on the fact that a responsive cell is defined by a peak that is more than 2-fold above mean baseline values obtained before stimulation. The first peak width was defined as the length of time between the first point where nuclear fluorescence was ≥2-fold above baseline and the subsequent time point when nuclear fluorescence fell 2-fold below that of baseline.

### Human Intestinal Tissue Specimens

Human biopsies (sigmoid colon and terminal ileum) obtained at colonoscopy following informed consent were stored at −80°C. After thawing on ice, they were lysed in 50 μL sterile PBS by high-speed shaking [2 × 2 min at 30 Hz; TissueLyser II (QIAGEN)]. After centrifugation at 10,000 × *g* for 15 min, each cleared tissue lysate was stored at −80°C.

### Cytokine Measurements

PBMDMs were stimulated with 200 ng/mL LPS for 20 h and culture medium was harvested and stored at −80°C for cytokine quantification. Cytokines were measured using the V-PLEX Proinflammatory Panel 1 Human kit (Meso Scale Discovery; Rockville MD, USA). Total protein was measured in biopsy lysates and PBMDM cultures using the bicinchoninic acid (BCA) assay (Pierce).

### Statistical Analysis

Mann-Whitney-Wilcoxon tests and Pearson's chi-squared proportional test were performed to compare patient demographics. *T*-tests and correlational tests were performed to compare the luciferase activation to clinical parameters. *P*-values were adjusted for multiple testing of outcomes using the false discovery rate method. Multivariate analyses were performed using the R language, and univariate analyses were performed using either the R language or GraphPad Prism v7.0. For cluster analysis, K-medoids algorithm was applied to stratify the patients with respect to their log_2_ fold-change of luciferase activity (31). The number of clusters was optimized with the average silhouette width criterion. In order to understand whether the association between disease status and luciferase activity was real, or confounded by other clinical or demographic factors, we performed a linear regression analysis of key variables against luciferase activity. Mann-Whitney *U*-test was used for univariate analysis of cytokine levels in LPS-stimulated PBMDMs, in serum and in biopsy lysates. Fisher's exact test was used to analyse the responding cells obtained by the confocal imaging data. Kruskal-Wallis test was used to analyse the peak width in the responding cells obtained by the confocal imaging data.

## Results

### Patient Demographics of the Main Cohort Study to Examine NF-κB Activity

As part of the SysMedIBD project (https://www.sysmedibd.eu/) a novel cohort of patients was recruited from two centers in Western Europe. In this study, we have investigated NF-κB signaling in 65 subjects, including healthy donors (Control) and patients with CD or UC (Table 1). UC patients were significantly older than those with CD (*p* = 0.03; Mann-Whitney-Wilcoxon test) and Control subjects (*p* = 0.01) and had a higher BMI than patients with CD (*p* = 0.04). CD patients also had higher serum CRP concentrations than those with UC (*p* = 0.03). No differences were detected in terms of smoking status, gender, use of immunomodulators (thiopurines, methotrexate), disease activity or biologics (infliximab, adalimumab, vedolizumab, or ustekinimab).

**Table 1:** Participants for NF-κB-regulated luciferase activity-based screening studies were recruited from outpatient clinics at The Royal Liverpool and Broadgreen University Hospitals NHS Trust, UK and University Hospital Aachen, Germany.

**Disease status**	**Crohn's disease (CD)**	**Ulcerative colitis (UC)**	**Control (Con)**	***p*-value CD vs. UC**	***p*-value CD vs. Con**	***p*-value UC vs. Con**
Number of patients	26	14	25			
Gender (% male)	50% (13/26)	64.3% (9/14)	40% (10/25)	0.594	0.663	0.262
Age (y)–Mean (SD)	38.3 (14.1)	48.5 (14.1)	35.3 (12.3)	0.033	0.534	0.010
BMI–Mean (SD)	24.2 (3.2)	28 (5.3)	27.6 (6.7)	0.044	0.229	0.966
**Smoking (%)^a^**						
Current	30.8% (4/26)	7.7% (1/13)	12.5% (3/24)	0.227	0.224	1
Previous	15.4% (8/26)	15.3% (2/13)	20.8% (5/24)	1	0.895	1
Never	53.8% (14/26)	77% (10/13)	66.7% (16/24)	0.295	0.525	0.783
CRP–Mean (SD)	7.3 (14)	1.7 (3.6)	2.1 (3.5)	0.031	0.799	0.088
Immunomodulator (thiopurines,methotrexate) %	23.1% (6/26)	14.3% (2/14)		0.803		
Biologic drug prescription (infliximab, adalimumab, vedolizumab, ustekinimab) %	30.8% (8/26)	28.6% (4/14)		1		
**Disease behavior (%)^b^**						
Penetrating disease (B3)	24% (6/25)					
Stricturing disease (B2)	24% (6/25)					
Neither stricturing, nor penetrating disease (B1)	52% (13/25)					

*BMI, Body mass index; CRP, C-reactive protein; SD, standard deviation*.

a *Missing data for one Ulcerative colitis (UC) patient and for one Healthy control (Con)*.

b *Missing data for one Crohn's disease (CD) patient*.

### Measurement of NF-κB Activity in PBMDMs Is Reproducible and Demonstrates Inter-individual Variability

**Figure 1:**
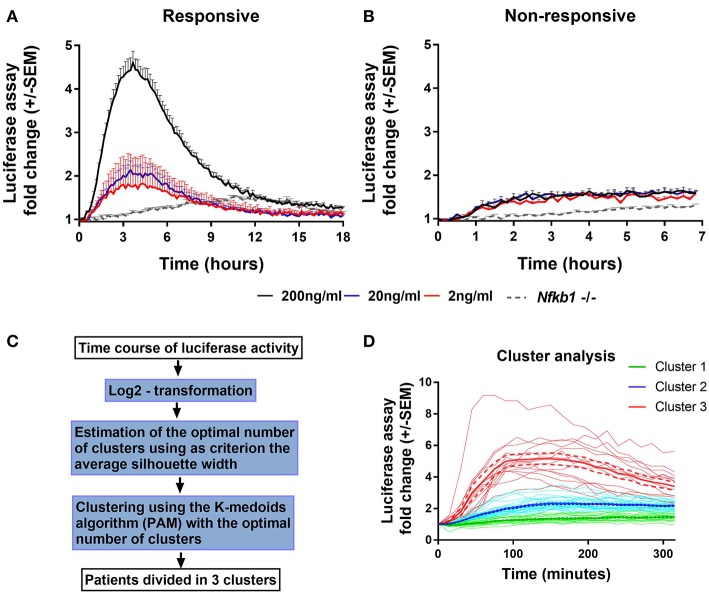
Endogenous NF-κB-regulated luciferase activity in lipopolysaccharide-stimulated PBMDMs. Graphs of κB-NLSluc luciferase activity measured over time (0–18 h) from differentiated human PBMDMs of two representative patients that were either **(A)** responsive or **(B)** non-responsive to stimulation with increasing doses of lipopolysaccharide (LPS); 2 ng/mL (red line), 20 ng/mL (blue line), and 200 ng/mL (black line); *n* = 5 vials PBMDM cultures for each patient. *Nfkb1*^−/−^ murine BMDMs (*N* = 3 mice, *n* = 2 replicates) stimulated with 200 ng/mL LPS were used as a negative luciferase activity control (gray dotted line). **(C)** Flow chart describing the steps that were followed for the clustering analysis (PAM = partitioning around medoids). **(D)** LPS-stimulated PBMDMs could assigned to one of three clusters based on log_2_ fold change in the luciferase activity profile using K-medoids algorithm. Bold lines show mean values, Dashed lines show ± standard error of the mean (SEM), and faint lines show individual cultures.

To characterize NF-κB activation in macrophages from our main cohort of patients and donors, isolated monocytes from PBMC samples were differentiated into PBMDMs and subsequently transduced with a lentiviral construct that expresses firefly luciferase under the control of the classical NF-κB promoter. Cultures were activated by addition of LPS, and luciferase activity was monitored over time. Normalization of luciferase activity toward the number of lentiviral copies in each culture was applied in order to consider possible differences in lentiviral transduction. To investigate the dynamics of this assay, we performed LPS dose-response studies. These demonstrated a strong luciferase response to LPS, at a dose of 200 ng/mL. BMDMs from *Nfkb1*^−/−^ mice, stimulated with 200 ng/ml LPS, were used as negative control because this strain lacks the p50 subunit and has impaired activation of the classical p50/p65 heterodimer downstream of TLR activation. As shown in Figure 1A, in the absence of the p50 subunit, no luciferase activity was detected, confirming the detrimental effect of p50 deletion on NF-κB-induced luciferase expression. However, marked differences in luciferase response were observed between individuals (Figure 1).

To determine whether this phenotypic variation was attributable to an extra-corporeal effect, we undertook validation studies in which we used multiple vials (*n* = 5) of PBMCs isolated from the same individual. Cells from each vial were thawed every week and differentiated, transduced and stimulated independently (Figures 1A,B). These data demonstrated reproducibility of luciferase activation plots, suggesting that the differences observed between samples from individuals represent biological differences, rather than being attributable to differences in culture technique.

To determine whether the luciferase response was affected by alternative sources of LPS, we used κB-NLSluc-transduced PBMDM samples from 12 randomly selected individuals from the CD cohort and stimulated them with doses of either commercial LPS from *Salmonella* Minnesota, or with LPS extracted from two CD mucosa-associated adherent, invasive *E. coli* strains, LF10 and LF82. All sources of LPS, tested at 200 ng/mL, induced a similar luciferase response (Supplementary Figure S1A). In addition, we also investigated the impact of various other stimulatory ligands on luciferase activity: Muramyl-dipeptide (MDP; an activator of nucleotide-binding oligomerization domain-containing protein 2, NOD2), flagellin (for TLR5), and IL-1β (for IL-1R), all induced NF-κB activation, but none of these stimuli were found to be as potent as LPS (Supplementary Figure S1B).

Having demonstrated the reproducibility of luciferase response to LPS, we screened PBMDMs derived from all patients to determine their luciferase responses. Patients were stratified according to their log_2_ fold change of luciferase activity expressed as area under the curve, using K-medoids algorithm to assign each sample to one of three predicted clusters, as it shown in Figure 1C. Cluster 1 contained samples from the least responsive group. Cluster 3 contained samples from the most responsive group, whilst Cluster 2 contained the samples that fell between these two extremes (Figure 1D). Samples from both Liverpool and Aachen were represented in each cluster and cells from healthy donors from both locations showed similar behaviors (Supplementary Figure S1C).

To visualize the dynamics of NF-κB activation, we used a lentivirus system to express human p65-AmCyan in primary PBMDM and determined the p65 nuclear translocation dynamics in freshly isolated cells from 14 individuals, by confocal microscopy (Supplementary Figure S2A). In resting cells, p65-AmCyan was localized almost exclusively in the cytoplasm within all cells but exhibited rapid nuclear translocation within minutes of stimulation (Supplementary Figures S2B–E). Out of >200 single cells that were assayed across all conditions ~40% of cells responded to Lipid A stimulation across individuals in the control group, although a significantly higher proportion of cells (61%) were responsive in the CD patient group; *p* < 0.001; two-sided Fisher's exact test by summation (Supplementary Figure S2F; Supplementary Table S1). Inspecting the characteristics of p65-AmCyan translocation in responding cells, we found that this did not differ between groups in terms of the average trajectory, timing and amplitude (Supplementary Figures S2G,H, respectively). The peak width, though, which reflects the duration of NF-κB nuclear localization, was observed to be significantly lower in responding cells from UC patients compared to either controls or CD patients (Supplementary Figure S2I; *p* < 0.001; Kruskal Wallis test).

### High Luciferase Activity Reflects a Strong Pro-inflammatory Phenotype

**Figure 2:**
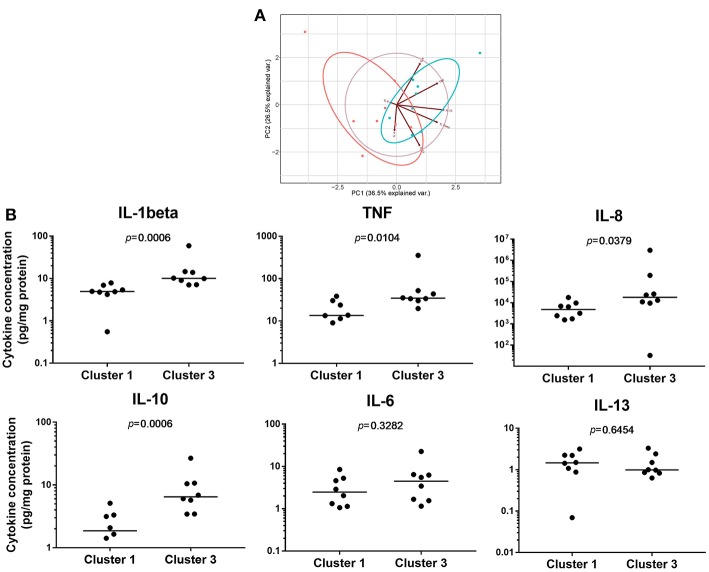
Pro-inflammatory cytokine levels can reflect differences between NF-κB-regulated luciferase activity defined clusters. **(A)** Principal component analysis (PCA) based on concentrations of pro-inflammatory cytokines secreted to the medium of 200 ng/mL lipopolysaccharide (LPS)-stimulated patient PBMDMs from Cluster 1 (red), and Cluster 3 (blue) samples. **(B)** Quantification of individual cytokines levels (pg/mL) released from patient PBMDMs over 20 h stimulation with LPS. Statistical comparisons between clusters were made using the Mann-Whitney *U*-test. Individual *p*-values are reported on each chart, with differences considered significant when *p* < 0.05.

To investigate the biological consequences of the differences in NF-κB activation observed, we measured pro-inflammatory cytokine concentrations produced by LPS-induced PBMDMs. For this purpose, we used samples from the two extreme clusters, Cluster 1 and Cluster 3, excluding further analysis of Cluster 2 which contained samples with luciferase activities of a highly variable nature. For this purpose, we identified 8 individuals from both Cluster 1 and Cluster 3 who had also undergone colonoscopy and biopsy of areas of macroscopically normal tissue from both the terminal ileum and sigmoid colon. New cultures of PBMDMs were prepared from each individual and these were stimulated with 200 ng/mL LPS for 20 h. The culture medium was harvested to measure the concentrations of several pro-inflammatory cytokines. Our initial analysis of this data was to perform a principal component analysis (PCA) and this demonstrated a clear separation between Cluster 1 and Cluster 3 based on stimulated cytokine secretion (Figure 2A). The differences demonstrated by PCA analysis are also reflected in analysis of the concentrations of individual cytokines. PBMDMs from Cluster 3 produced substantially higher levels of TNF (*p* = 0.01; Mann-Whitney *U*-test), IL-1β (*p* < 0.001), IL-10 (*p* < 0.001), and IL-8 (*p* = 0.04) compared to Cluster 1 (Figure 2B) by univariate analysis. When corrections were made for multiple testing using the FDR method, the differences observed for TNF, IL-1β, and IL-10 remained statistically significant.

To determine whether these differences in cytokine production were also reflected in the serum or enteric mucosa of the patients from whom the PBMDMs had been derived, we performed cytokine assays using serum, as well as homogenized tissue lysates from the terminal ileum and sigmoid colon of the same patients (Supplementary Figure S3). In the serum, IL-1β levels were undetectable (dynamic range 0.05–375 pg/mL), but the concentrations of the other cytokines that were examined were found to be similar between the two clusters. Similarly, few differences were observed in cytokine levels in intestinal tissue homogenates between patients from each the two cluster. The only exception was a marginal, but significantly lower abundance of IL-13 observed in the lysates of sigmoid colon tissue obtained from patients in Cluster 3 (Cluster 1; median 9.9 pg/mL (IQR, 7.4-11.4) vs. Cluster 3; median 5.3 pg/mL (IQR, 4.4–7.5); *p* = 0.0401, Mann-Whitney *U*-test). However, this difference was not statistically significant when *p*-values were corrected for multiple comparison by FDR. Further comparison of the levels of each cytokine from LPS-stimulated PBMDMs (dynamic measurement) and intestinal tissue samples (static measurement) also revealed no correlation between dynamic and static measurements (Supplementary Figure S4). This suggests that the dynamic measurement of *in-vitro* cytokine production likely reflects the increased activity of endogenous NF-κB and the pro-inflammatory status of the PBMDMs.

### Low NF-κB Regulated Luciferase Activity Is Characteristic of Ulcerative Colitis (UC) Patients

**Figure 3:**
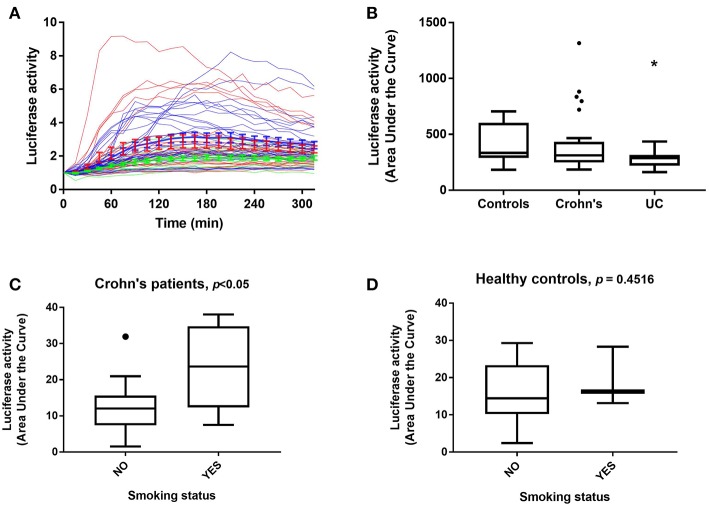
NF-κB-regulated luciferase activity in healthy control and IBD patient donors. Luciferase activity from all patient PBMDMs screened represented as **(A)** a dynamic, color-coded graph of activity over time in response to 200 ng/mL LPS (blue, healthy controls; red, Crohn's disease (CD) and green, ulcerative colitis (UC), and as **(B)** area under the curve (AUC) for control, CD, and UC groups. NF-κB-regulated luciferase activity in **(C)** CD patients, and **(D)** healthy control donors based on smoking status. Statistical comparisons between disease types were made using the Kruskal-Wallis test, *p < 0.05. Statistical differences between smoking status were tested using the Mann-Whitney *U*-test, individual *p*-values are reported on each chart.

Within the three distinct clusters representing differential luciferase activity, we noticed that the majority of UC patients were assigned to Cluster 1. We therefore analyzed luciferase activity data obtained from patient samples taken from both clinical sites (Liverpool and Aachen) and based on disease status (Figures 3A,B). A marked difference in luciferase activity from PBMDMs in response to stimulation with 200 ng/mL LPS over 20 h was observed only between 24 healthy donors (controls) and 14 UC samples (and not with CD), with UC samples showing significantly lower levels of luciferase activity; *p* < 0.05, Kruskal-Wallis test (Figure 3B and Supplementary Figure S5). By contrast, PBMDMs derived from the 26 CD patients were represented in all three assigned clusters, with a very broad spectrum of luciferase activity being observed upon stimulation with LPS. Correlation between the disease status and clusters revealed that the percentage of UC patients decreased within clusters associated with higher NF-κB activation (Cluster 1: 34.78, Cluster 2: 20.68, and Cluster 3: 0%) (Supplementary Figure S5). In contrast, the percentage of CD patients positively correlated with increased luciferase activity, especially observed in Cluster 3. Further analysis of the metadata available for 24 of the CD patients, revealed that PBMDMs from these patients who were active smokers (*N* = 6) showed significantly higher luciferase activity than those from non-smokers (*N* = 18) (Figure 3C; *p* < 0.05, ANOVA). Interestingly, this was not the case for the healthy donor control sample LPS-stimulated PBMDMs, where there was no observed difference between smokers (*N* = 3), and non-smokers (*N* = 21); *p* = 0.4516 (Figure 3D). Most patients with UC are non-smokers, hence only 1 sample was from a UC patient who was a current smoker.

### Smoking Status Is the Only Independent Factor Which Correlated With Luciferase Activity

In this relatively small cohort, our analysis demonstrated that non-smokers had substantially lower luciferase activity than current smokers (coefficient: 7.68, 95% confidence interval: 1.53 to 13.83, *p* = 0.015) (Table 2). No differences were detected in terms of gender, or use of immunomodulators (thiopurines, methotrexate) or biologics (infliximab, adalimumab, vedolizumab or ustekinimab). Patients with UC also had a trend toward lower luciferase activity than control patients, but this just failed to reach statistical significance (*p* = 0.070). There was no significant difference between recruitment centers (*p* = 0.771), and neither gender (*p* = 0.678), age (*p* = 0.585), nor concomitant immunomodulatory (*p* = 0.978), and/or biologic drug use (*p* = 0.972), influenced the outcome of the luciferase activity assay. C-reactive protein (CRP) levels identified from the metadata, although higher in the CD group, did not correlate with the patient clusters and therefore macrophage-specific luciferase activity.

**Table 2:** Demographic characteristics and associations with the Area under the curve (AUC) of the NF-κB-regulated luciferase activity.

**Variable**	**Coefficient**	**95% Confidence intervals**	***p-*value**
**Center**			
Aachen vs. liverpool	−0.78	(−6.16; 4.59)	0.771
**Diagnosis**			
CD vs. Con	−4.37	(−11.17; 2.43)	0.202
UC vs. Con	−6.77	(−14.13; 0.58)	0.070
Sex (male vs. female)	0.93	(−3.54; 5.40)	0.678
Age (in years)	0.05	(−0.13; 0.22)	0.585
**Smoking**			
Current vs. never	7.68	(1.53; 13.83)	0.015
Previous vs. never	0.43	(−5.74; 6.59)	0.890
Behavior CD (B2/B3 vs. B1)	3.84	(−4.24; 11.93)	0.344
Immunomodulators (yes vs. no)	0.10	(−6.90; 7.10)	0.978
Biologics (yes vs. no)	−0.13	(−7.43; 7.18)	0.972

*Linear regression was performed using 60 patients with complete metadata; Crohn's disease (CD), ulcerative colitis (UC), and healthy control donors (Con). Each coefficient was tested with t-test. For the categorical variable diagnosis, healthy controls were taken as reference. For the categorical variable smoking, never smoking was taken as a reference. For the categorical variable behavior CD, the categories B2 and B3 were grouped together, and tested against the reference B1*.

## Discussion

Inflammatory bowel disease has complex pathogenesis involving genetic susceptibility, intestinal microbiota, the host immune system and environmental factors such as diet, stress, smoking, and hygiene (32). Defects in signaling pathways can lead to dysregulation of the inflammatory response that are crucial in the pathogenesis of IBD. Genome-wide association studies have identified numerous genetic loci associated with risk for IBD and quantitative studies have also shown differential gene expression in the innate immune cells (monocytes, macrophages) from IBD patients (33–36). One of the most studied pathways is the NF-κB signaling pathway which was first linked to IBD in 1998 (21). Since then, many laboratories have shown hyper-activation of the NF-κB signaling pathways in intestinal epithelial or immune cells from IBD patients (11, 21, 37–39).

In this study, we designed a screening strategy of human PBMDMs based on an NF-κB-regulated luciferase reporter *in vitro* assay. Luciferase reporter assays are widely used because they are convenient, relatively inexpensive, and give quantitative measurements instantaneously (40). Frozen, human PBMCs were used as starting material, and following successful optimization of the macrophage culture and lentivirus infection conditions we developed a robust luciferase assay protocol that achieved highly reproducible results. Moreover, no luciferase activity was detected when the assay was tested on *Nfkb1*^−/−^ murine macrophages that lack the p105/p50 subunit, showing that this assay can reflect genetic defects in the NF-κB pathway. In our screen, control healthy donors from both clinical sites, Liverpool and Aachen, showed similar luciferase profiles which could reliably be used for comparison with samples from individuals affected by IBD. Luciferase activity profiles were further analyzed and used to cluster the samples into three groups, with Cluster 1 defined as a low activity group and Cluster 3 as a high activity group. Individuals with CD showed a broader spectrum of luciferase activity, but their percentage in clusters positively correlated with increasing NF-κB-regulated luciferase activity. On the other hand the majority of UC patients were assigned to Cluster 1, whereas no UC patients were assigned to Cluster 3 which represents the highest NF-κB-regulated luciferase activity. This observation is however different from previous reports of high NF-κB activity in UC patients (11, 21). This discrepancy may be due to the cell types that have been tested, as well as the procedures that were used to prepare and assay the samples. In future studies, it would be of great interest to assess NF-κB-regulated luciferase activity in specific intestinal mucosal macrophage populations, such as those found in lamina propria. However, there are limitations to this approach. As discussed in a recent review, the resident intestinal macrophages are typically of low yield showing great heterogeneity, consisting of short-lived and long-lived populations, each with variable phenotype. Moreover, in IBD, the dynamics of blood monocyte differentiation into resident intestinal macrophages is likely altered (41). It has also been shown recently that the macrophage-monocyte balance within the colon is also altered during colitis, with pro-inflammatory blood monocytes increasingly recruited to the lamina propria (42).

Many cytokines are regulated by the same signaling pathways and NF-κB is a major pro-inflammatory transcription factor in immune cells (43). We therefore compared cytokine levels induced by LPS stimulation from cells of individuals in Cluster 1 and Cluster 3. Those data taken together with luciferase activity responses, showed that there was a positive correlation between cytokine levels and endogenous NF-κB activation in human PBMDMs. Static measurements of cytokines in either matched intestinal biopsies or serum did not however demonstrate any correlation with NF-κB based clustering or cytokine production.

Confocal microscopy has previously been used extensively in cell lines and primary cells for quantitative measurement of NF-κB nuclear translocation (8, 44–46) and here we were also successful in visualizing the NF-κB/p65 activation dynamics in freshly isolated human PBMDMs. Under our experimental conditions, the p65 subunit exhibited rapid nuclear translocation, upon stimulation and this was similar to previous analyses of murine bone marrow-derived macrophages (9, 10). Interestingly, differences were observed in the percentage of responding cells (higher in CD patients) and in the peak width (shorter in UC patients). The number of responding cells could play an important role *in vivo*, where macrophage response can affect the local environment. The peak width defines the kinetics of NF-κB dwell time in the nucleus and we have shown previously that inhibition of nuclear export affects the dynamics of p65 localization as detected by confocal imaging (24). This defines cell specific patterns of gene expression (4, 47) and could correlate to disease status. This is the first report that has indicated a correlation between NF-κB activation and IBD in primary human PBMDMs by confocal imaging. However, due to the small sample size and complexity of disease, it is not possible to make firm conclusions and further experiments will be necessary to validate our imaging observations.

Multivariate analysis of the entire cohort revealed that there was no correlation between luciferase activity and demographic and clinical data, such as age, sex, CRP levels, or use of immunomodulators or biologics. Although UC patients studied here were 10–13 years older than CD and controls, it has been shown previously that macrophages and dendritic cells show no difference in response to LPS stimulation (and subsequent TLR4 receptor signaling) with increasing age of individuals (48). Whilst CRP levels were also noted to be higher in our CD patient cohort compared to those with UC, suggesting perhaps an important difference in the inflammatory status among patients, CRP increases are indicative of an acute phase inflammatory response and provide only a limited reflection of disease activity (or severity) in CD (49). We also observed that there was no correlation of LPS-induced PBMDM luciferase response with patient disease activity or severity. This was not surprising though as previous research has shown that the phenotype of *in vitro* activated PBMCs does not reflect disease activity of IBD patients (50). Metadata analysis of our main cohort of patients with CD, revealed that samples from active smokers had statistically significant higher NF-κB activity compared to non-smokers within this disease subgroup. This difference was not observed in the healthy control donors who smoked. Moreover, multivariate analysis of the entire cohort revealed that the only independent factor that predicted differences in luciferase activity was smoking, whereas age, sex, use of immunomodulators or biologics had no effect. There is an established strong association between smoking and CD, perhaps best demonstrated in the recent TOPPIC trial which identified smoking as the only factor which predicted post-operative recurrence in CD (51). Moreover, there is a differential effect of smoking on gastrointestinal inflammation in CD patients compared to UC (52). Our data raises the hypothesis that the association between smoking and CD disease may be due to an effect of this combination on NF-κB signaling dynamics. Whilst we did not directly investigate the mechanisms that underlie links between smoking and NF-κB activation, we have shown that differences in our assay are sustained even after prolonged storage of cells, suggesting that it is unlikely to be a short-term influence of specific components of cigarette smoke that influences the differences we observe. Cigarette smoking is reported to have long term epigenetic effects, some of which are permanent, whilst others are reversible after smoking cessation (53). This mechanism may be one way by which smoking can have a sustained influence of NF-κB activity *in vitro*.

Amongst the top 10 research priorities identified by a recent James Lind alliance priority setting partnership was “*What are the optimal markers/combinations of markers (clinical, endoscopic, imaging, genetics, other biomarkers) for stratification of patients with regards to a) disease course, b) monitoring disease activity and c) treatment response?*” (54). The luciferase assay we describe here shows promise both as a potential predictor of diagnosis of UC, and also as a potential stratification tool for future therapeutic studies that propose to target NF-κB activity. Before these uses can be established however, there will need to be a more extensive assessment of the assay in larger cohorts and these studies will need to be appropriately powered to determine how effectively the assay functions. A personalized medicine approach for IBD is attractive and our screening assay, described in this study appears to be able to segregate IBD patients and therefore has the potential to be used for further *in vitro* drug testing, specifically for patients who do not respond to therapy. An additional, and more pragmatic future goal may also be to use this assay in combination with epigenetic studies in an attempt to identify links between IBD, NF-κB activation and smoking status. Smoking-induced cell-specific epigenetic modifications could affect pathways, such as the NF-κB, in ways that could explain differential response to environmental factors, as well as to therapeutic approaches.

## Data Availability

All datasets generated for this study are included in the manuscript/Supplementary Files.

## Ethics Statement

The studies involving human participants were reviewed and approved by Patient and healthy control blood samples and intestinal tissue biopsy specimens were obtained following informed consent and with study approvals from NRES Committee North West-Liverpool East (R&D 4910; REC 15/NW/0045) and the Regional Human Ethics Committee, Aachen, Germany (EK 235/13). The patients/participants provided their written informed consent to participate in this study.

## Author Contributions

SP, HE, and MB: acquisition of data. SP, MB, FB, and HE: analysis and interpretation of data. FB and BC: data analysis, bioinformatics, and statistical analysis. RH: human sample preparation and isolation of PBMCs. LS: lentiviral preparation. DS: confocal imaging. BC: purification of LPS from clinical *E. coli* isolates. CP, DP, DJ, and GS: provision of patient samples. SP, MB, and BC: drafted manuscript. MW, BC, CP, DJ, GS, VM, PP, RH, WM, and DP: critical revision of the manuscript for important intellectual content. MW, BC, DP, VM, DJ, CP, and WM: obtained funding. All the authors approved the final manuscript submission.

### Conflict of Interest Statement

VM is a director and shareholder of LifeGlimmer GmbH. FB has received a salary from LifeGlimmer GmbH. The remaining authors declare that the research was conducted in the absence of any commercial or financial relationships that could be construed as a potential conflict of interest.
