# Supplementary material for: Macrophage-Specific NF-κB Activation Dynamics Can Segregate Inflammatory Bowel Disease Patients
Source: Front Immunol. 2019 Sep 11;10:2168. doi: 10.3389/fimmu.2019.02168 (PMC6749845; doi:10.3389/fimmu.2019.02168)
Supplement: Supplementary file 1 [file Data_Sheet_1.PDF]

Supplementary information

Macrophage-specific NF-κB activation dynamics can segregate inflammatory bowel disease patients

Stamatia Papoutsopoulou, Michael D. Burkitt, François Bergey, Hazel England, Rachael Hough, Lorraine Schmidt, David G. Spiller, Michael H.R. White, Pawel Paszek, Dean A. Jackson, Vitor A.P. Martins Dos Santos, Gernot Sellge, D. Mark Pritchard, Barry J. Campbell, Werner Müller and Chris S. Probert

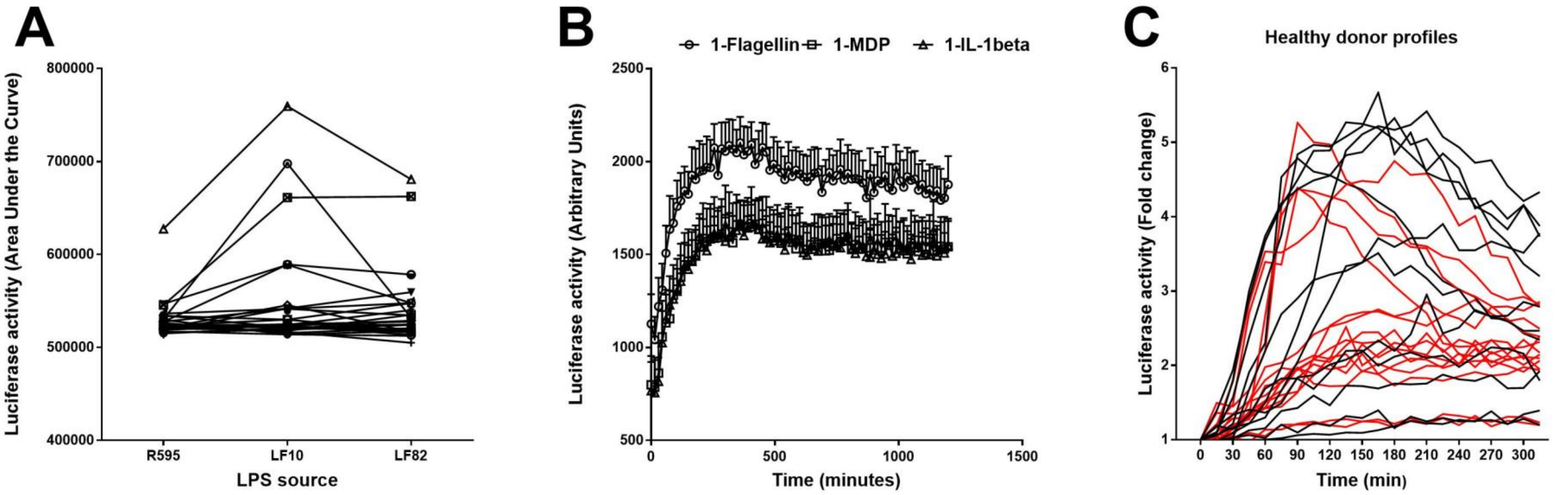

Figure S1: Luciferase activity of PBMDMs stimulated by various ligands. PBMDMs show similar NF-κB luciferase activity, represented as area under the curve, as a response to 200ng/mL LPS from various sources (A). PBMDMs were stimulated with 100ng/mL Flagellin (o-), 500ng/mL MDP (-□-) and 20ng/mL IL-1β (-△-) and luciferase activity was measured over time, as shown in (B). Luciferase activity profiles in LPS-stimulated PBDMs from healthy donors from Liverpool (black) and Aachen (red) (C).

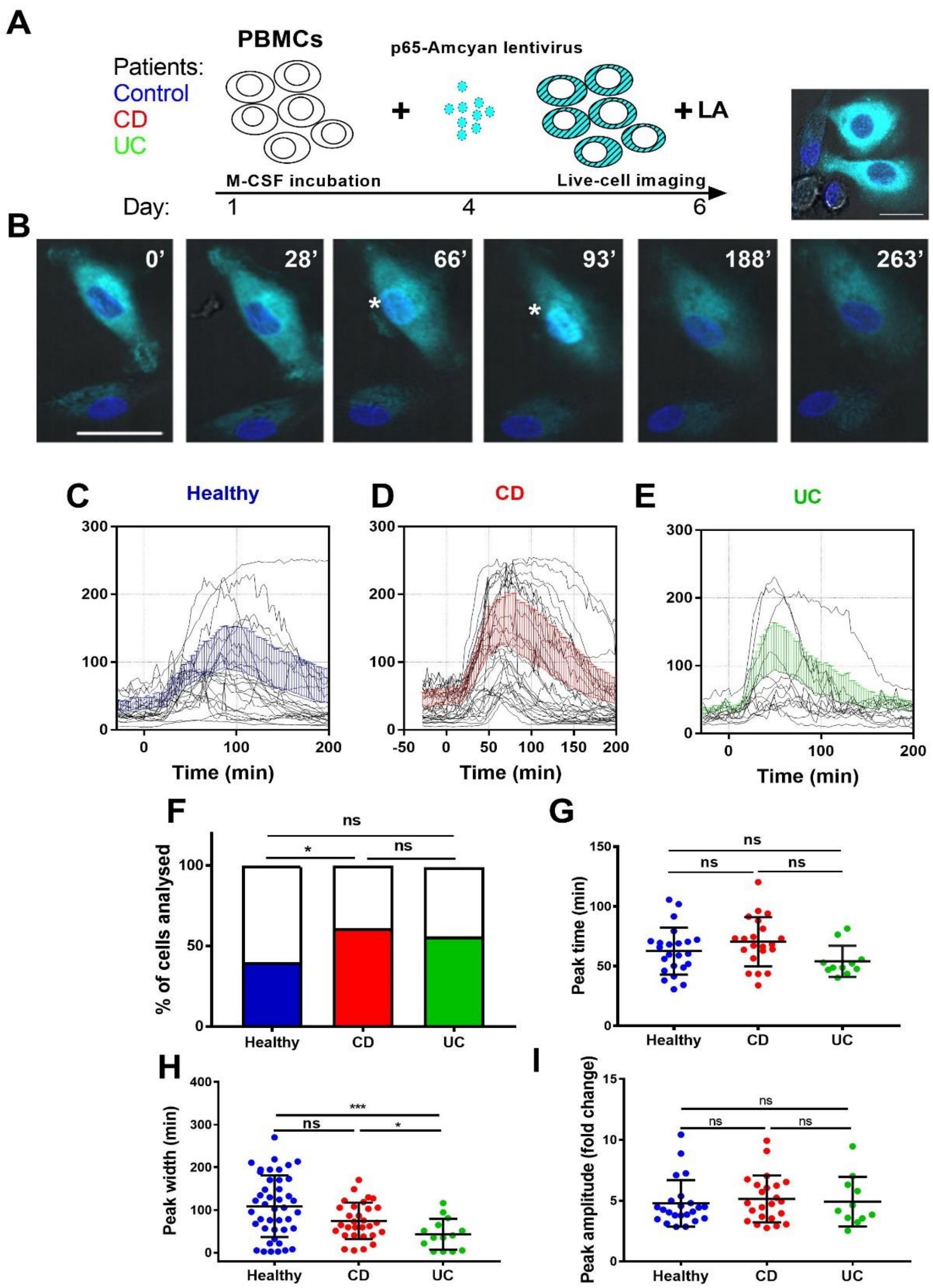

Figure S2: Time-lapse Confocal imaging of NF-κB nuclear translocation in Lipid A-stimulated human PBMDMs. (A) PBMC-derived macrophages from human subjects were transduced with a fluorescent lentiviral p65AmCyan construct and were used to image NF-κB p65 subunit cellular dynamics in real-time. (B) CellTracker was used to generate profiles of nuclear translocation of transduced cells post stimulation with 200ng/mL Lipid A, over 3h: Asterisks (*) indicate nuclear localization of p65 at 66 and 93 min (‘). (C-E) Graphs of individual nuclear translocation profiles of responding cells in the healthy controls, Crohn’s disease (CD) and ulcerative colitis (UC) groups; mean ± SD for all responsive cells shown in blue, red and green colour lines for each group, respectively. (F) The proportion of responding cells was calculated by pooling all tracked cells from each disease group; *p<0.05; two-sided Fisher’s exact test (by summation). Peak identification and analysis revealed in each population the peak time (G), peak amplitude (H) and peak width (I): *p<0.05, ***p<0.001; statistical differences assessed by Kruskal Wallis test with pairwise comparison of all three groups.

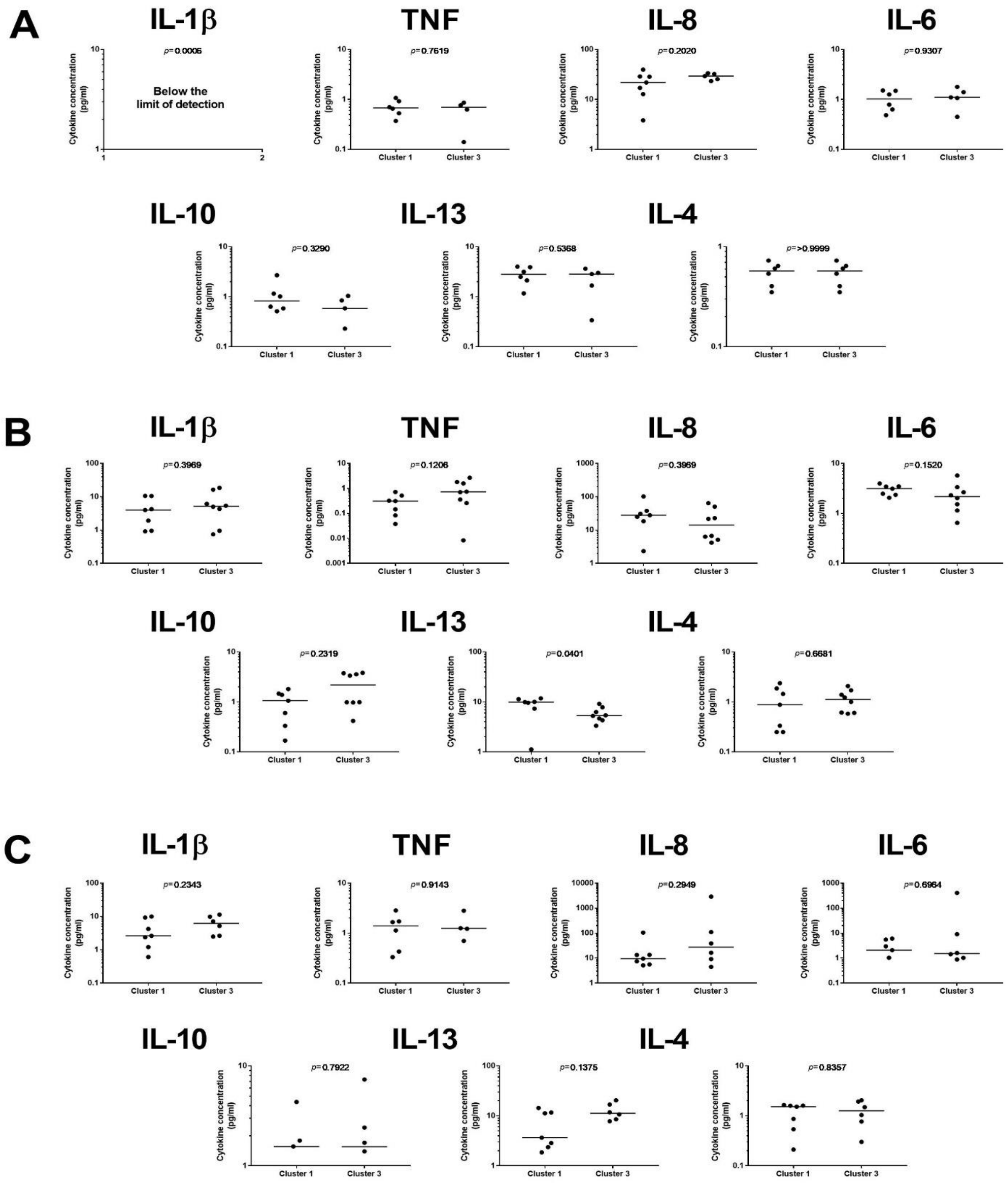

Figure S3: There is no detectable difference in cytokine levels between Clusters 1 and 3 in serum or colonic biopsies from IBD patients. Measurement of proinflammatory cytokine levels in serum (top panel), sigmoid colon biopsy lysates (middle panel) and terminal ileum biopsy lysates (bottom panel). Total protein was quantified in the lysates and used for normalisation of the ELISA data.

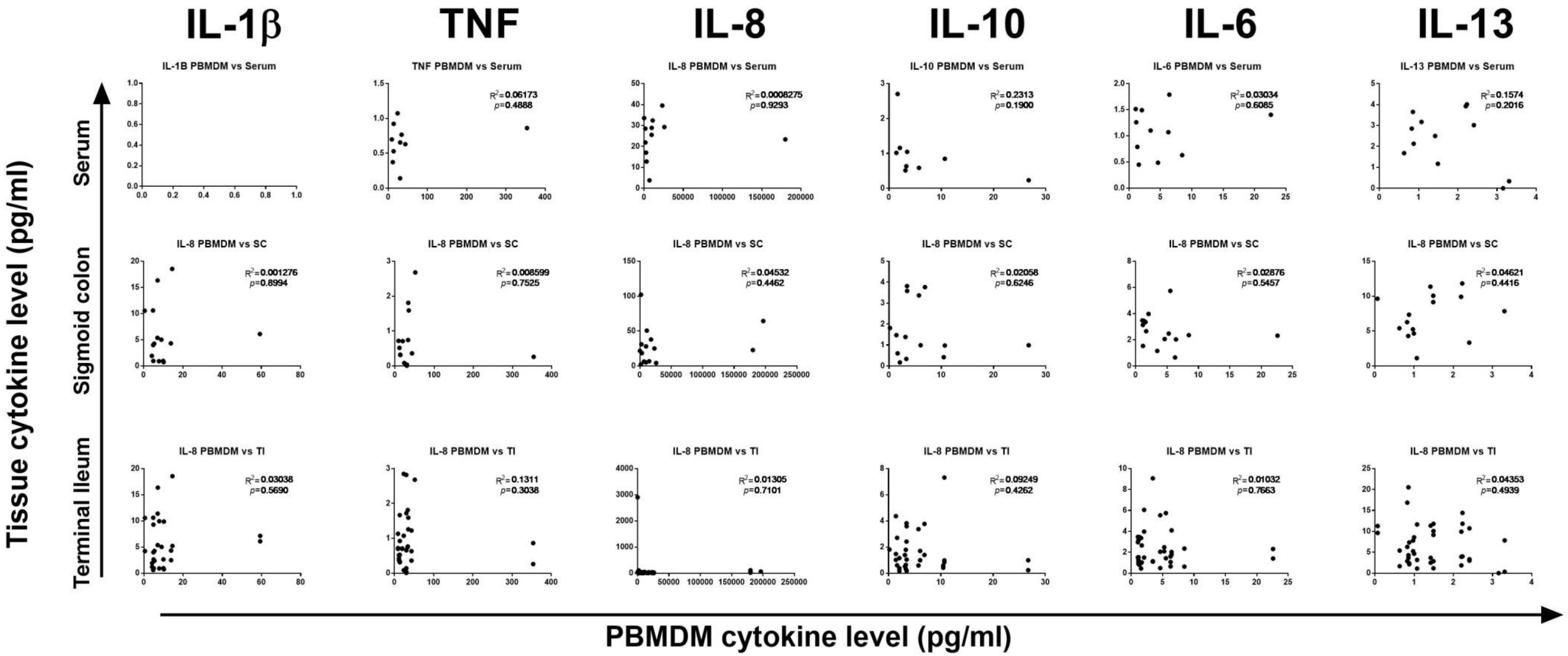

Figure S4: There is no correlation between dynamic and static measurements in cytokine levels. Comparison of cytokine levels produced by LPS-stimulated PBMDMs (dynamic) versus cytokine levels detected in serum (top panel), sigmoid colon biopsy lysates (middle panel) and terminal ileum biopsy lysates (bottom panel). Results of sum-of-squares linear regression analysis (r2 and F-test p- values) are reported on each graph.

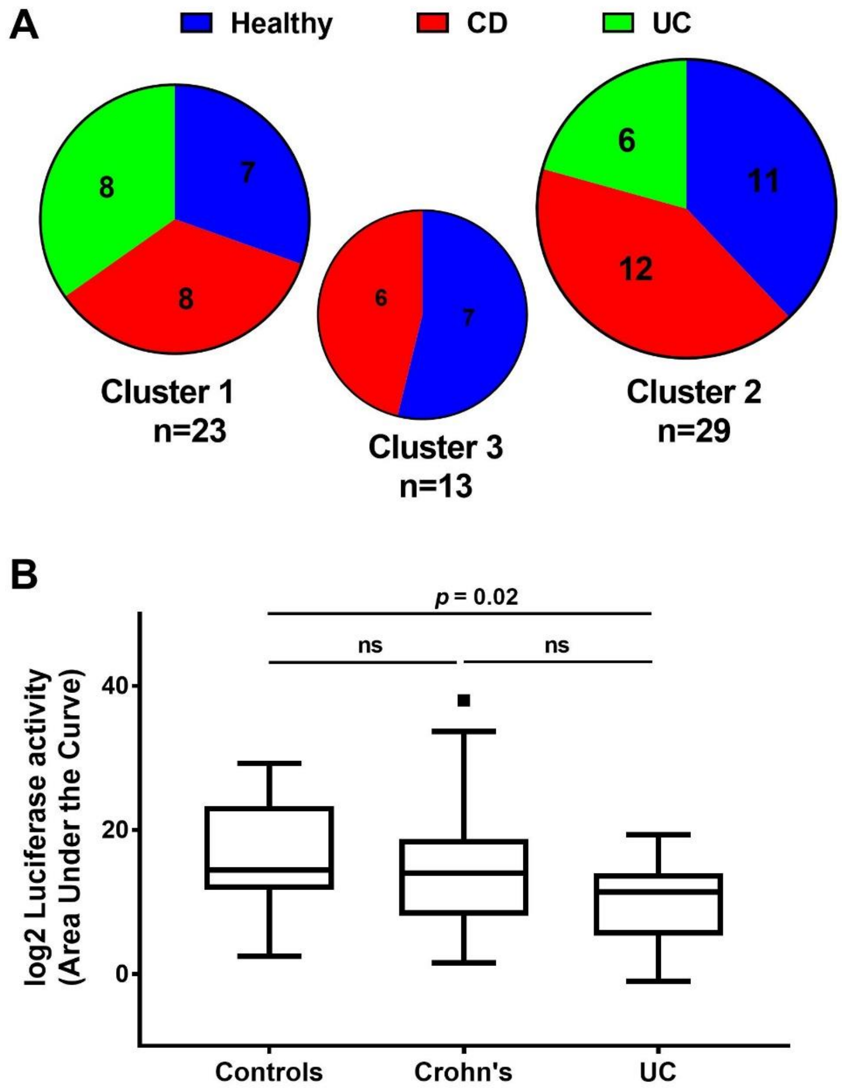

Figure S5: Clustering analysis of luciferase activity. (**A**) Pie charts representing each cluster, the total number of patients as well as the numbers of Control, UC and CD patients within each cluster. (**B**) Luciferase activity from all patient PBMDMs screened represented as log_2 area under the curve (AUC) for control, CD and UC groups. Statistical comparisons between disease types were made using the Kruskal-Wallis test.

Supplementary Table S1: **Percentage of responding cells in samples from each patient as assessed by confocal imaging of PBMDM cultures infected with lentivirus vector expressing the human p65-AmCyan protein and stimulated *in vitro* with 200ng/mL Lipid A.**

**Healthy controls**<br><br>**N=6**	**CD**<br><br>**N=5**	**UC**<br><br>**N=3**
25.0	16.7	60.0
70.4	33.3	20.0
30.0	85.7	90.0
30.0	63.6	
10.0	66.7	
57.1		

The total percentage of responding cells was calculated based on the assumption that a responsive cell is defined by a peak that is more than 2-fold above baseline values (i.e. mean of the values before stimulation). CD = Crohn's disease, UC = ulcerative colitis
